# Polysaccharides from *Paecilomyces hepiali* Prevent Acute Colitis in Association with Modulating Gut Microbiota and Treg/Th17 Immune Balance in Mice

**DOI:** 10.3390/molecules28134984

**Published:** 2023-06-25

**Authors:** Luwen Cui, Ningning He, Shengnan Yu, Hao Pang, Zixuan Zhang, Jingyi Wang, Jianhua Hao, Shangyong Li

**Affiliations:** 1 School of Basic Medicine, Qingdao University, Qingdao 266071, China; clwdyouxiang@163.com (L.C.); heningning@qdu.edu.cn (N.H.); yushengnan2020@163.com (S.Y.); ph17561673629@163.com (H.P.); zzx15505490861@163.com (Z.Z.); jywyouxiang2023@163.com (J.W.); 2 Key Laboratory of Sustainable Development of Polar Fishery, Ministry of Agriculture and Rural Affairs, Yellow Sea Fisheries Research Institute, Chinese Academy of Fishery Sciences, Qingdao 266071, China; 3 Laboratory for Marine Drugs and Bioproducts of Qingdao National Laboratory for Marine Science and Technology, Qingdao 266000, China

**Keywords:** cordyceps polysaccharide, *Paecilomyces hepialid*, ulcerative colitis, gut microbiota, intestinal homeostasis

## Abstract

Cordyceps exopolysaccharide (CEP) has shown emerging potential in adjustment of gut microbiota and immune cell function. In this study, a water-soluble CEP with a molecular weight of 58.14 kDa was extracted from the fermentation broth of *Paecilomyces hepiali*, an endophytic fungus of Cordyceps sinensis. Our results indicated that *Paecilomyces hepiali* polysaccharide (PHP) showed significantly preventive potential on dextran sulfate sodium (DSS)-induced colitis in mice, which can prevent colon shortening, reduce intestinal epithelial cell (IEC) destruction, suppress inflammatory cell infiltration, and regulate the balance between regulatory T (Treg) cells and T helper type 17 (Th17) cells. Meanwhile, the disturbed gut microbiota was partially restored after PHP treatment. Further Pearson correlation coefficient analyses exhibited that the alteration of the gut microbiota was significantly related to adjustment of the IEC barrier and Treg/Th17 balance. In conclusion, all findings proposed that purified PHP has the potential to develop into a promising agent for colitis prevention and adjuvant therapy via maintaining intestinal homeostasis of gut microbiota and immune system.

## 1. Introduction

Ulcerative colitis (UC) is a chronic, progressive, and potentially disabling inflammatory bowel disease with clinical manifestations of bloody diarrhea, abdominal pain, and emergency, and the majority of patients have a higher risk of relapse [1,2,3]. With a global incidence of 7.6–246 people per 100,000 individuals, UC has now emerged as a tough challenge for global public health [2]. Nevertheless, the available drugs, including 5-aminosalicylic acid (5-ASA), glucocorticoids, immunosuppressant agents, and monoclonal antibodies against relevant cytokines, cannot effectively prevent recurrence and sustain remission of UC [4]. Existing IBD treatment intervention drugs also include new strategies, such as monoclonal antibodies, which block pro-inflammatory cytokines (tumor necrosis factor-α (TNF-α)), sphingosine 1-phosphate inhibitors, and the JAK kinase inhibitors [5].

UC has debatable etiology, and is closely related to genetic and homeostatic environmental changes, gut microbiota, and mucosal immune system dysfunction [6]. Gut microbiota is thought to be one of the key factors in regulating host health, and is related to host protective immunity and epithelial barrier functioning [7,8]. In UC patients, impaired epithelial barrier functioning allows normally harmless symbiotic bacteria to cross the epithelium and become pathogenic bacteria, causing the immune system to attack and drive them out, thereby exacerbating intestinal inflammation [9]. Hitherto, the interventions adjusting the structure of gut microbiota, functional foods as prebiotics, and healthy donor fecal microbiota transplantation (FMT) are some of the current and futuristic methods for the UC adjunctive therapy [6]. Different nutritional plans and dietary supplements can lead to changes in the composition and function of gut microbiota, thereby affecting the progression of UC [10,11]. In recent years, natural polysaccharides (including pectin, guar gum, rhamnogalacturonan, chitosan, fructan, psyllium, glycosaminoglycan, algal polysaccharides, polysaccharides from fungi and traditional Chinese medicine, and degraded polysaccharides from *Porphyra haitanensis*) have become a research hotspot due to their therapeutic effects on UC [12,13]. These effects are related to the regulation of inflammatory factors, gut microbiota, the immune system, and the protection of intestinal mucosa.

Cordyceps sinensis as a parasitic edible ascomycetous fungus have been applied extensively in traditional Chinese medicine with various biological and pharmacological functions [14]. Its main active ingredient is Cordyceps exopolysaccharide (CEP), which provides a good prospective application in the prevention and treatment of many diseases [15]. Due to many factors, the sustainable utilization of natural Cordyceps sinensis resources have been seriously threatened, which lead to over-digging [16]. *Paecilomyces hepiali* is a type of Cordyceps fungus that can produce CEP by large-scale fermentation, which has been widely used in clinical and health foods. *Paecilomyces hepiali* polysaccharide (PHP) showed various bioactivities, such as anti-fatigue and anti-hypoxic effects [17], anti-diabetic and anti-nephritic effects [18], and anti-tumor and antioxidant activity [19,20]. However, it is still unclear whether PHP has a preventive and therapeutic effect on inflammatory bowel disease, and whether PHP has similar efficacy against native CEP in UC treatment, and its structure–activity relationship is unclear.

In this study, our results indicated that PHP showed potential anti-inflammatory and gut microbiota regulating effects in a dextran sulfate sodium (DSS)-induced mouse colitis model, which make PHP a promising curative agent for prevention and adjuvant therapy of UC by maintaining gut homeostasis.

## 2. Results

### 2.1. Structure Characterization of PHP

**Figure 1:**
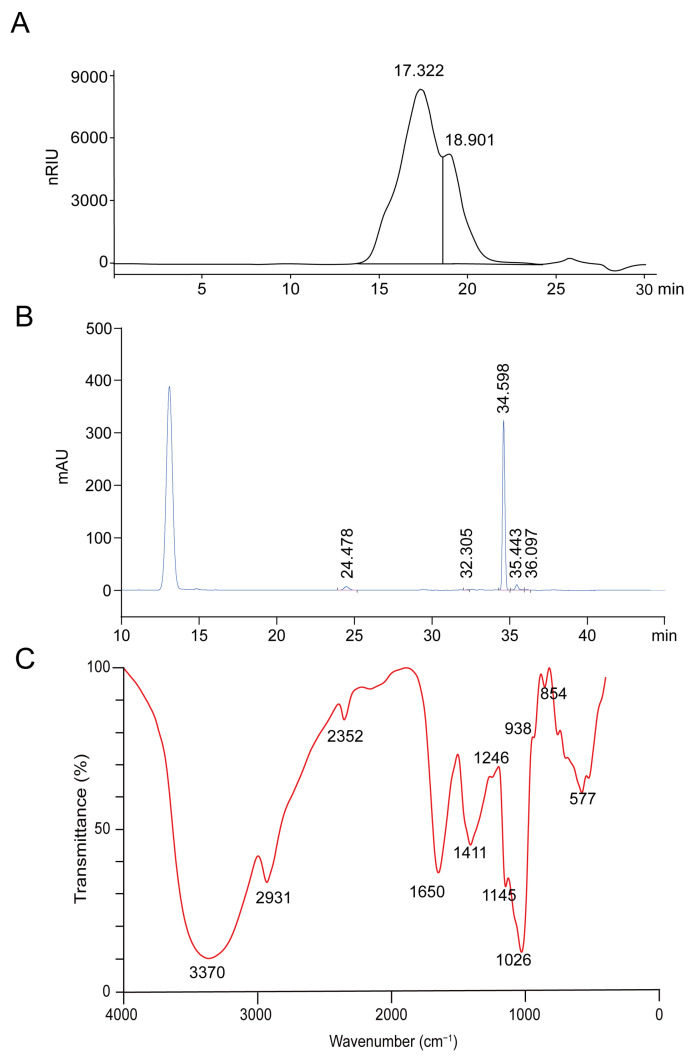
Primary structural characterization of PHP. The MW (**A**) and monosaccharide composition (**B**) determinates of PHP, normalized response integral units (nRIU) and milli−absorbance unit (mAU). (**C**) FT-IR spectrum of PHP.

Commonly, the pharmacological activity of polysaccharides usually depends on the structure of polysaccharides and other physicochemical properties, including MW, monosaccharide composition, uronic acid content, etc. [21]. To better comprehend the biological activity of PHP, we further explored its structure characterization. After analysis, the MW of PHP was 58.14 kDa (Figure 1A). The monosaccharide composition of PHP was presented in Figure 1B. PHP mainly contained D-mannose (2.49%), glucose (57.1%), galactose (1.43%) and D-galacturonic acid (0.321%) (Figure 1B). FT-IR spectrum was shown in Figure 1C. The absorption peak at 3370 cm^−1^ was corresponding to the stretching vibrations of O−H, whereas the weak band at 2931 cm^−1^ was assigned to stretching vibrations of C−H [22]. The absorption peak at 1650 and 2352 cm^−1^ were related to vibration of C=O bonds [23], and the spectra of PHP have peaks at 1411, 1246 and 938 cm^−1^, which may put down to ester and carboxylate groups [24]. 

### 2.2. PHP Supplement Effectively Alleviates DSS Induced Symptoms

**Figure 2:**
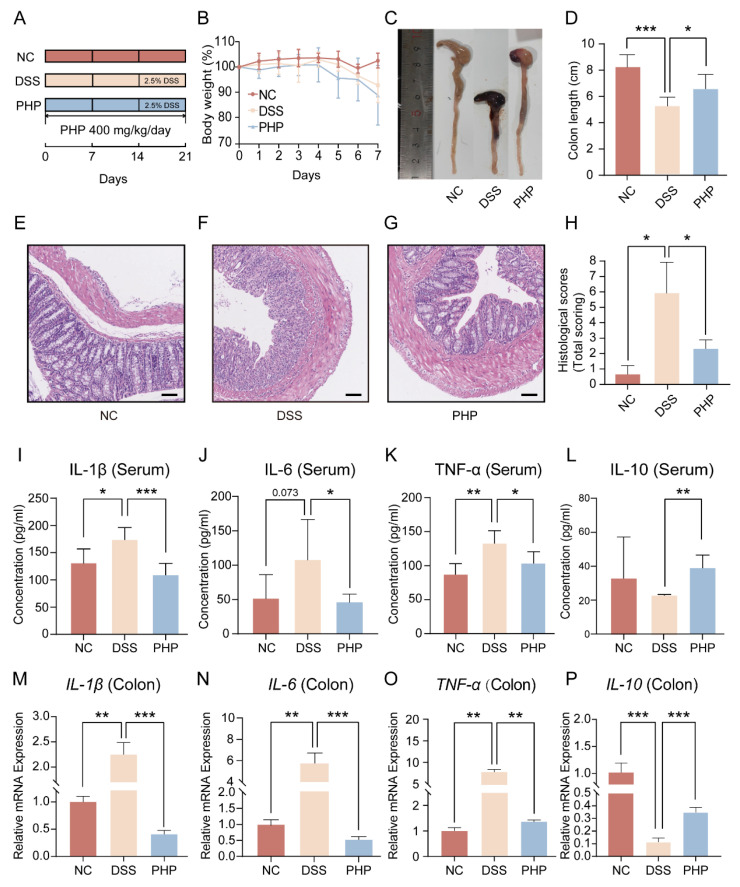
PHP significantly alleviated the DSS-induced colitis in mice. (**A**) The flow chart of experimental design (NC or PHP: n = 6, DSS: n = 7). (**B**) The changes in body weight. (**C**) Images of colon. (**D**) The quantification of the colon length. (**E**–**G**) Representative images of the H&E-stained colon sections. The plotting scale = 100 μm. (**H**) Histological scores of colons (n = 3). The concentrations of IL-1β (**I**), IL-6 (**J**), TNF-α (**K**) and IL-10 (**L**) in serum. The relative mRNA expression levels of *IL-1β* (**M**), *IL-6* (**N**), *TNF-α* (**O**) and *IL-10* (**P**) in colon tissue. Compared with the DSS group, * *p* < 0.05, ** *p* < 0.01, and *** *p* < 0.001.

In order to study the anti-colitis role of PHP, the DSS-induced colitis mouse model was constructed (Figure 2A). Obviously, the body weights of mice in the DSS group were significantly decreased after DSS treatment, but PHP treatment did not improve the weight of mice significantly (Figure 2B). Colon length is usually used as one of the key macroscopic indicators to assess the severeness of colitis, and its length is inversely proportional to the severeness of colitis. As shown in Figure 2C,D, the colon length of DSS group was significantly lower than the NC group (*p* < 0.001), while the colon shortening caused by DSS was reversed after PHP supplement (*p* < 0.05). In order to further study the protective role of PHP on the colon, H&E staining was carried out on colon sections of mice (Figure 2E–H). Compared to the NC group, the histopathological examination of the DSS group showed more inflammatory cell infiltration, glandular loss, and mucosal epithelial necrosis (Figure 2E,F). In contrast, the pathological injury of the colon was restored by PHP supplementation (Figure 2G). Furthermore, histological scores of colon mucosa showed that PHP had a therapeutic effect on colon injury (Figure 2H). According to the above results, PHP has a good protective role on alleviating the pathological damage of the colon caused by DSS. Moreover, proinflammatory and anti-inflammatory factors play an important part in the immune response of UC. It was found that the nature of the mucosal immune response was determined by proinflammatory and anti-inflammatory factors, and the imbalance between them was a critical factor of abnormality of the mucosal immune response [25]. To further evaluate the impact of PHP on intestinal inflammatory and systemic response, anti-inflammatory and proinflammatory factors in the colonic tissues and serum were measured (Figure 2I–P). Compared with the DSS group, the concentrations of proinflammatory cytokines (IL-6, TNF-α and IL-1β) in the PHP group were decreased, while the concentration of anti-inflammatory factor (IL-10) was increased in serum (Figure 2I–L). Meanwhile, RT-qPCR was used to confirm the gene expression of cytokines associated with inflammation (*IL-10, TNF-α, IL-6 and IL-1β*) in the colon tissues, which was in accord with the trend of ELISA (Figure 2M–P). Taken together, these results collectively showed that PHP can inhibit DSS-induced colitis symptoms, colon injury and inflammatory reaction.

### 2.3. PHP Helps Maintain the Integrity of the Intestinal Barrier

**Figure 3:**
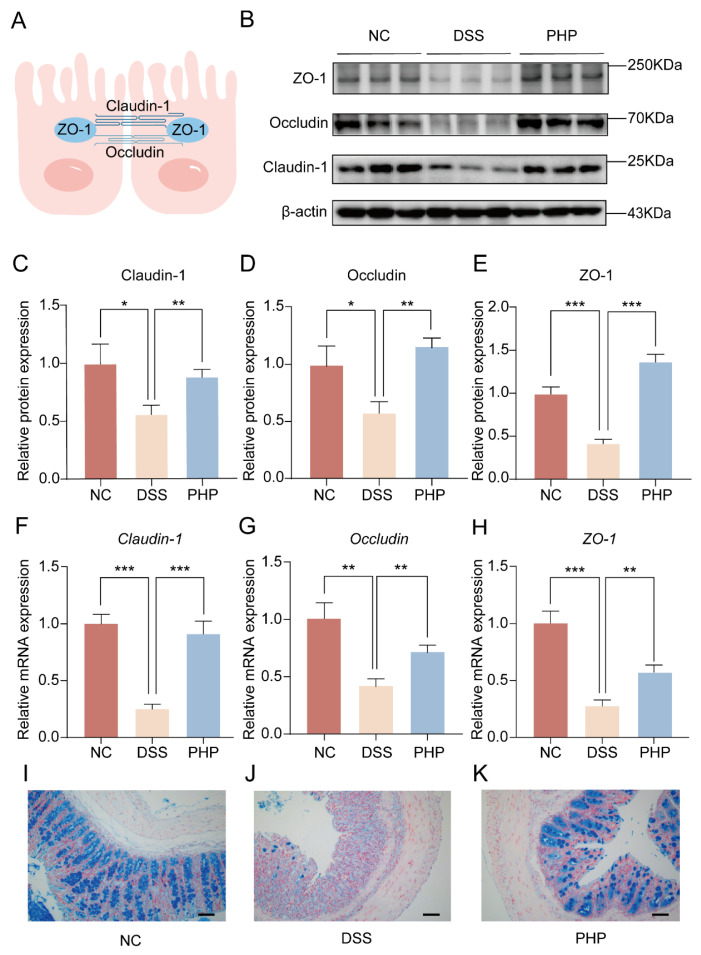
Effects of PHP on the functioning of the intestinal barrier. (**A**) Mechanism diagram of TJ proteins. (**B**) WB analysis of ZO-1, Occludin and Claudin-1 (n = 3). The quantitative analysis of protein expressions of Claudin-1 (**C**), Occludin (**D**) and zonula occludens-1 (ZO-1) (**E**). The relative mRNA expressions of *Claudin-1* (**F**), *Occludin* (**G**) and *ZO-1* (**H**). (**I**–**K**) Representative images of alcian blue staining of colon sections. Plotting scale = 50 μm. Compared with the DSS group. * *p* < 0.05, ** *p* < 0.01, *** *p* < 0.001.

Tight junction (TJ) proteins (ZO-1, Occludin and Claudin-1) play an import part in the maintenance of intestine barriers to prevent the transmission of potentially harmful pathogens and toxins [26] (Figure 3A). Herein, the regulative actions of PHP on intestinal TJ proteins were measured by RT-qPCR and WB (Figure 3). DSS induction significantly reduced the protein and gene expressions of these TJ proteins, while PHP treatment increased the protein and gene expression of TJ proteins (Figure 3B–H). These results showed that PHP treatment could significantly reverse the decrease of TJ proteins expression caused by DSS destruction, thereby enhancing the change of epithelial permeability and the stability of epithelial barrier structure. Moreover, the colonic mucus layer was studied utilizing alcian blue staining to better realize the impact of PHP on mucosal barrier functions (Figure 3I–K). It can be seen that the secretion defects caused by the disappearance of goblet cells and their secretory vesicles resulted in the reduction in mucus production in the DSS group (Figure 3J). Interestingly, mucosal changes were reversed to a large extent in the PHP group (Figure 3K). Above all, these results delineated that PHP improves intestinal injury, possibly by promoting the expression of TJ proteins and the function of mucosal barriers.

### 2.4. PHP Improves Treg/Th17 Cell Balance to Regulate Immunity

**Figure 4:**
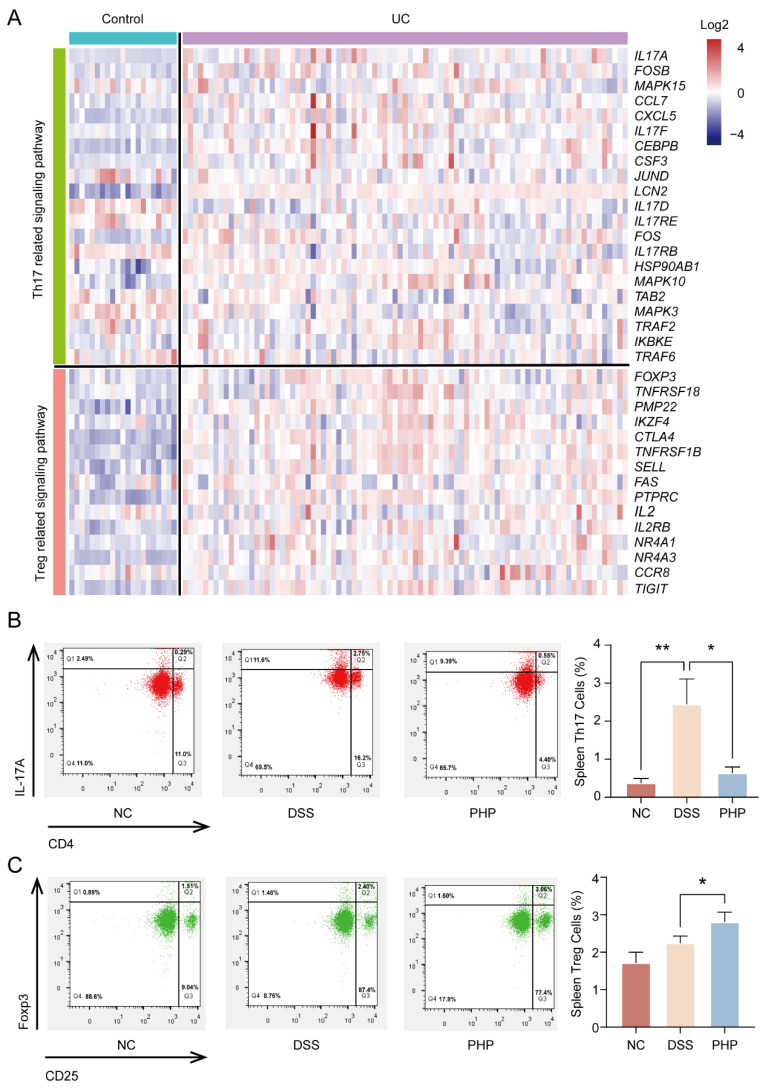
PHP regulates the Treg/Th17 balance in DSS-induced colitis mice. (**A**) The gene expression pattern of Th17 and Treg pathway related genes in UC and healthy samples from GEO database. Red represents high expression and blue indicates low expression. (**B**) Th17 (CD3^+^CD4^+^IL17A^+^) cells and (**C**) Treg (CD3^+^CD4^+^CD25^+^Foxp3^+^) cells in the spleen (n = 3) were analyzed by flow cytometry analysis. Compared with the DSS group, * *p* < 0.05, ** *p* < 0.01.

It was discovered that the balance of Treg/Th17 cells is essential for the regulation of UC treatment [27,28]. The change of characteristic genes belonging to Treg/Th17 pathways were displayed via analysis of transcriptome data of colitis tissues from UC patients and healthy individuals in the GEO database (Figure 4A). The findings demonstrated that there were significant differences in different gene expression patterns of Treg/Th17 pathways between the UC group and the control group, which further suggested that Treg/Th17 balance played an important role in the occurrence and development of UC. Using flow cytometry, the proportion of CD3^+^CD4^+^CD25^+^Treg^+^ and CD3^+^CD4^+^Th17^+^ cells in mice spleens was identified and analyzed to further investigate the impact of PHP on the balance of Treg/Th17 cells (Figure 4B,C). The results showed that, compared with NC group, the proportion of Treg and Th17 cells in DSS group changed, which was consistent with the results of the outcomes shown in Figure 4A. At the same time, compared with the DSS group, the number of Th17 cells in spleen lymphocytes of PHP group mice decreased significantly (*p* < 0.01), while the number of Treg cells increased significantly (*p* < 0.05) (Figure 4B,C). These findings illustrate that PHP could restore the intestinal immune function of DSS-induced mice by regulating the balance between Th17 and Treg cells.

### 2.5. PHP Alters the Relative Abundance of Gut Microbiota

**Figure 5:**
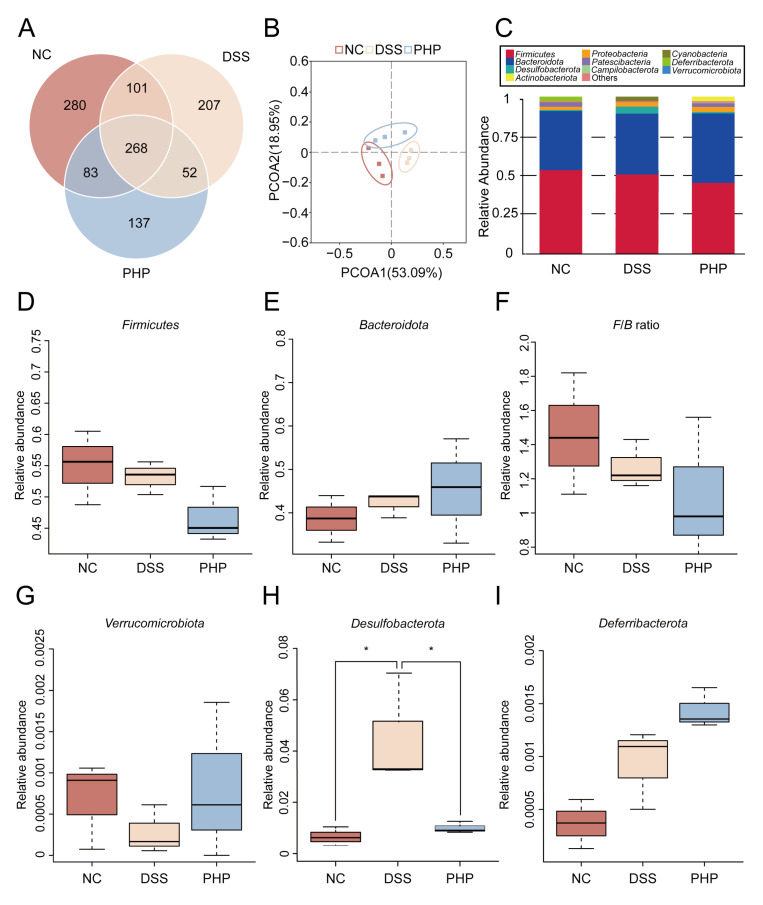
PHP adjusted the entirety construction of the gut microbiota. The fecal samples were collected for 16S rRNA sequencing (n = 3). (**A**) Venn diagram of OTUs. (**B**) Principal coordinate analysis (PCoA) plots upon the difference among three groups. (**C**) The phylum-level abundance of gut microbiota. The relative abundance of *Firmicutes* (**D**), *Bacteroidota* (**E**), F/B ratio (**F**), *Verrucomicrobiota* (**G**), *Desulfobacterota* (**H**) and *Deferribacterota* (**I**). All at the level of phylum, * *p* < 0.05.

To assess the alteration of gut microbiota in response to PHP, fecal samples from mice were tested by 16S rRNA sequencing. A Venn diagram describes the operational taxonomic unit (OTU) changes in the three groups (Figure 5A). It showed that 268 OTUs coexisted in 3 groups, 101 OTUs coexisted between the NC group and DSS group, 52 OTUs coexisted between the DSS group and the PHP group, and 83 OTUs coexisted between the PHP group and the NC group. Different OTU diversity in each group indicates that PHP treatment dramatically changed the composition of gut microbiota. There was no significant difference in α-diversity and β-diversity between the NC, DSS, and PH groups (data not shown). The structural changes of gut microbiota were analyzed by principal coordinates component analysis (PCoA) based on UniFrac distance. As can be seen in Figure 5B, when compared to the NC group, DSS administration dramatically altered the composition of the gut microbiota, whereas PHP treatment significantly reversed this trend, making it closer to the NC group. Meanwhile, we analyzed the relative abundance of three groups of gut microbiota at the phylum level (Figure 5C), and found that the PHP group had the highest relative abundance of *Actinobacteriota* among the three groups. 

The gut microbiota changed dramatically at the phylum level after PHP treatment. To further evaluate the relative abundance changes in the gut microbiota, we have selected some bacteria to separately describe relative abundance at the phylum level (Figure 5D–I). Compared with DSS group, the relative abundance of *Firmicutes* decreased remarkably in PHP group (Figure 5D), while the relative abundance of *Bacteroidota* increased to some extent (Figure 5E), resulting in a noteworthy reduction in the value of F/B (Figure 5F). Compared with the DSS group, the relative abundance of *Verrucomicrobiota* and *Deferribacterota* in the PHP group increased as the phylum level increases (Figure 5G,I), while the relative abundance of *Desulfobacterota* was significantly decreased (*p* < 0.05) (Figure 5H).

**Figure 6:**
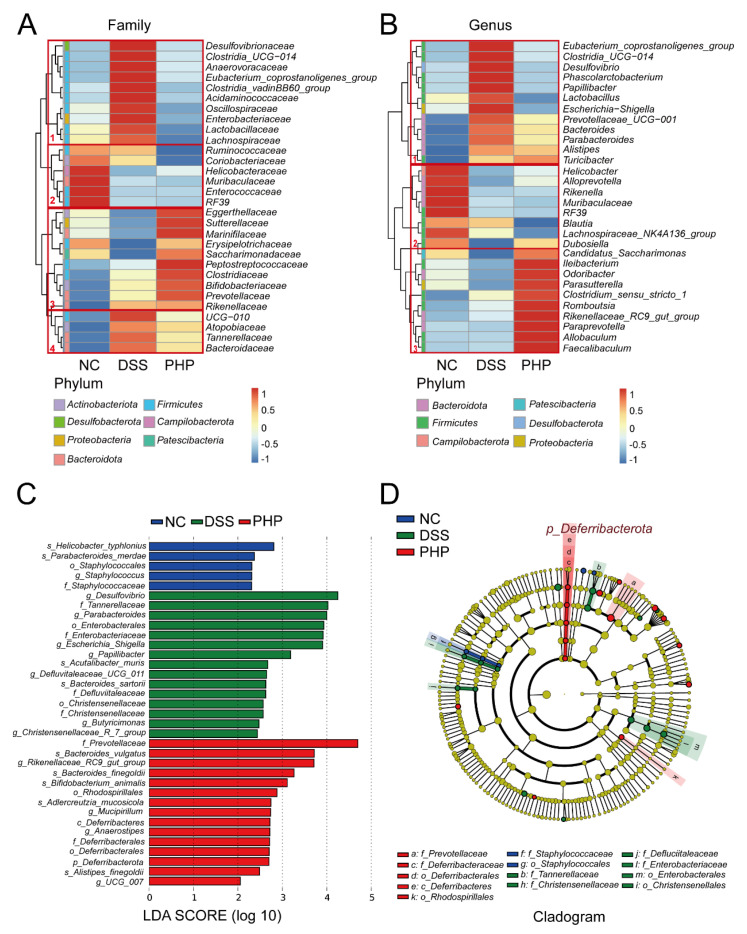
Alteration of gut microbiota by PHP. Heatmap depicted the normalized abundance of each microbiota from fecal samples among the NC, DSS and PHP groups at the family level (**A**) and at the genus level (**B**). The Y-axis distribution is divided into families or genera belonging to different phyla, and the X-axis shows the abundance distribution of different groups. (**C**) Distribution histogram acquired applying LEfSe in the NC, DSS, and PHP groups, *p* < 0.05 and LDA score (log_10_) > 2 displayed. (**D**) Cladogram illustrating the results of LEfSe analysis.

Meanwhile, a heat map further proved the differences at the family level and genus level in gut microbiota among the three groups (Figure 6A,B). At a family level, in cluster 1, *Desulfovibrionaceae, Anaerovoracaceae, Oscillospiraceae, Enterobacteriaceae, Lactobacillaceae* and *Lachnospiraceae* were significantly over-represented in the DSS group compared with the NC group, while this state was reversed after treatment of PHP (Figure 6A). The gut microbiota in cluster 1 in the DSS group was increased at the genus level (Figure 6B). However, after PHP supplementation, the bacteria in cluster 1 recovered to the NC level. This also significantly enhanced the abundance of gut microbiota in cluster 3, mostly the beneficial bacteria, such as *Faecalibacterium* and *Romboutsia* (Figure 6B). Linear discriminant analysis (LDA) effect size (LEfSe) analysis was used to identify the specific bacterial taxa characterized among each group (Figure 6C,D). Figure 6C,D shows the species with significant differences, indicated by an LDA score greater than 2.0, which mirrors the degree of influence of different treatments for gut microbiota. LEfSe analysis showed the LDA score of pro-inflammatory bacteria, such as *Desulfovibrio*, *Escherichia Shigella* and *Enterobacteriaceae* was greater than 3 in the DSS group (Figure 6C). Conversely, the PHP group had a lot of beneficial bacteria with higher scores, such as *Prevotellaceae*, *Bacteroides_vulgatus* and the *Rikenellaceae_RC9_gut_group* (Figure 6C). The cladogram in Figure 6D presented the markedly differential taxonomic features and their phylogenetic relationships. We found that *Deferribacteraceae* located within the phylum *Deferribacterota*, were the prominent species in the PHP group, and the family of *Enterobacteriaceae* was the main bacteria species of the DSS group (Figure 6D). Taken together, our results reveal the regulatory effect of PHP on gut microbiota.

### 2.6. Correlation Analysis of UC-Related Symptoms and Treg/Th17-Related Immunity with Gut Microbiota

**Figure 7:**
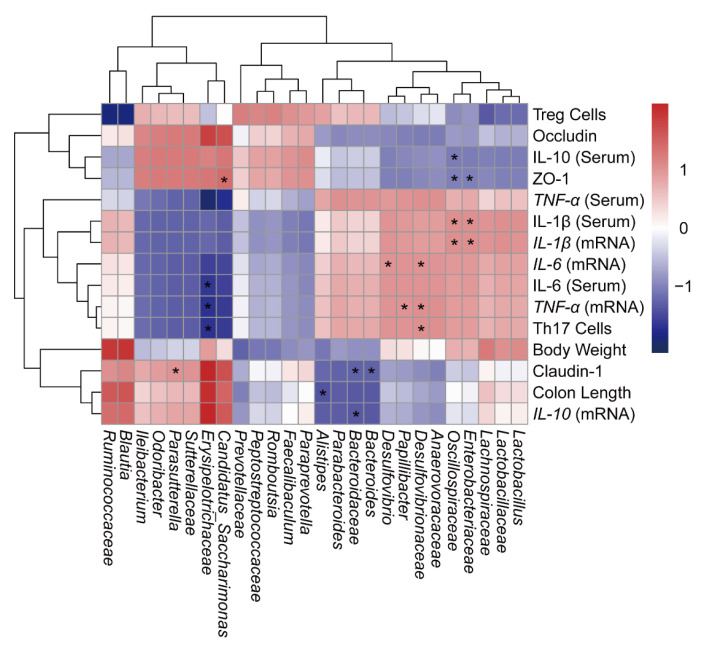
Pearson correlation analyses of relations between gut microbiota and UC-related symptoms or Treg/Th17-related immunity. The strength of the colors is proportional to the degree of association (red, positive correlation; blue, negative correlation). * *p* < 0.05.

In order to comprehensively analyze the relationship between UC-related symptom parameters and gut microbiota, the Pearson’s correlation coefficient was calculated to generate a correlation matrix (Figure 7). The results showed that there was a negative correlation between *Bacteroidaceae*, *Enterobacteriaceae* and *Oscillospiraceae* with TJ proteins (Claudin-1, Occludin and ZO-1). It was worth mentioning that *Candidatus Saccharimonas*, *Parasutterella* and *Erysipelotrichaceae* were positively correlated with TJ proteins, while negatively correlated with pro-inflammatory factors, suggesting that *Candidatus Saccharimonas*, *Parasutterella* and *Erysipelotrichaceae* had the potential to repair intestinal damage. Meanwhile, the abundance of *Erysipelotrichaceae* was negatively correlated with the number of Th17 cells. Above all, it was speculated that there was a connection between the alteration of different bacteria and the colitis-related parameters, indicating that there is a close relationship between the inflammatory response and immunity and the alteration of gut microbiota.

**Figure 8:**
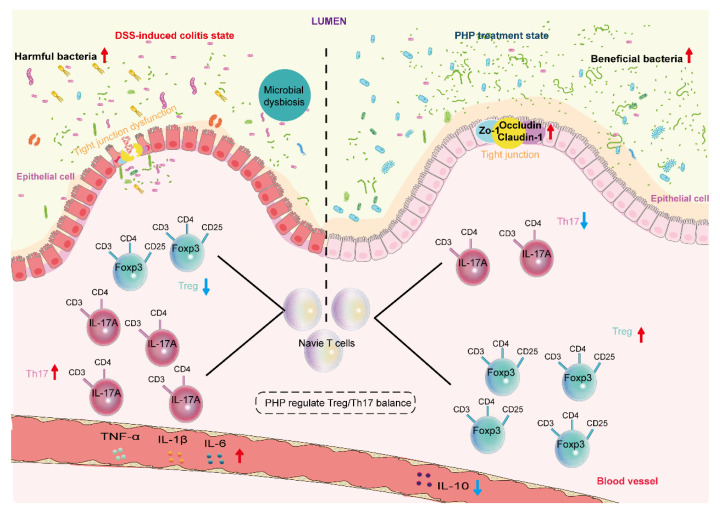
The proposed mechanism of PHP in preventing and treating DSS-induced colitis.

The above results suggest that the beneficial effect of PHP on colitis is mainly achieved by improving the imbalance of microbiota, maintaining the intestinal barrier function, and maintaining the balance of Treg/Th17 cells (Figure 8).

## 3. Discussion

As a traditional medicinal fungus, the quality and efficacy of natural Cordyceps sinensis harvested in different regions, altitude, and climate conditions vary greatly [29]. The main purpose of this study was to investigate the active polysaccharide for the treatment of DSS-induced colitis from industrial *Paecilomyces hepiali,* a Cordyceps fungus, and to probe the underlying mechanisms. We verified that PHP could enhance the integrity of intestinal epithelial barrier, reduce various inflammatory cytokines, regulate the Treg/Th17 imbalance, and ameliorate the dysbiosis of gut microbiota. These findings make PHP a suitable functional food for inhibiting inflammatory responses and regulating gut homeostasis.

The pathogenesis of UC is closely correlated with the damage of intestinal epithelial barrier structures and functions [30]. Intestinal epithelial cells connect with each other by TJ proteins, such as ZO-1, Claudin-1 and Occludin, forming a tight but selective barrier that allows electrolyte and nutrient absorption, provides energy for the growth of intestinal symbionts, and blocks lumen bacterial invasion [31,32]. Disturbance of intestinal TJ protein expression/localization leads to severe intestinal barrier damage, and bacterial and endotoxin invasion, which increases systemic inflammation and immune response [33,34]. Our results confirmed the good therapeutic effect of PHP on the intestinal barrier and tight connections destroyed by DSS in morphology (Figure 3E–G), and gene expression and protein analyses (Figure 4B–H), indicating its potential in the adjuvant therapy of UC.

In addition, Treg/Th17 imbalance plays an indispensable role in the pathogenesis of UC. Treg cells played a regulatory role in immunity by secreting IL-10 [35]. Th17 cells participate in, and promote the occurrence and development of, multiple autoimmune diseases, mainly producing pro-inflammatory cytokines [36,37]. It is worth noting that strongly inducing Th17 cell differentiation aggravated colitis in the mice model, while stimulating Treg cell differentiation could inhibit adaptive and innate immune responses to alleviate UC [38,39]. Therefore, ameliorating the Treg/Th17 balance is helpful to re-establish intestinal immune homeostasis [40]. In this study, PHP administration selectively upregulated the number of Treg cells and downregulated the proportion of Th17 cells, improving the Treg/Th17 balance in the DSS-induced UC model, which was consistent with the mechanism of Gegen Qinlian decoction [41] and Berberine [42] in treating UC.

Dysbiosis alterations of gut microbiota composition have long been intimately bound up with chronic inflammation, and are responsible for pathogenesis in colitis [43]. There is a widespread brief that most polysaccharides not digested by the small bowel perform their physiological functions through the metabolic breakdown of gut microbiota [43]. Therefore, we speculated that the gut microbiota might be responsible for the therapeutic effects for colitis of PHP and, subsequently, inspected the effect for PHP on the gut microbiota dysbiosis in DSS-induced colitis. In our study, we found that PHP treatment reversed the structure’s change of microbiota caused by DSS, augmented the abundance of beneficial microbiota, and lessened the abundance of harmful microbiota, hinting its potential prebiotics activity (Figure 5 and Figure 6). In Figure 5C, we observed that PHP can increase the abundance of *Actinobacteriota*. Some known *Actinobacteriota* can protect the health of the host by inhibiting the growth and proliferation of harmful bacteria, enhancing intestinal immunity and regulating the balance of gut microbiota in the intestine. For example, *Streptomyces*, *Actinomadura*, and *Micromonospora* have the ability to produce active metabolites such as antibiotics, cathelicidin, and acids, which can inhibit the growth of intestinal pathogens and alleviate intestinal inflammation [44,45]. The phylum Verrucomicrobiota, with potential anti-inflammatory properties, was significantly increased in the PHP group [46] (Figure 5G). Butyrate can be absorbed by colonocytes and gut microbiota as an energy source, which is an essential metabolite with an anti-inflammatory effect [47,48]. The intake of PHP could augment the abundance of butyrate-producing bacteria, including Faecalibaculum and Prevotellaceae [49,50] (Figure 6A,B). Furthermore, we found that supplementing with PHP remarkably reduced the relative abundance of Desulfovibrio compared with the DSS group (Figure 6B). The Desulfovibrio vulgaris can induce increased barrier permeability in polarized Caco-2 cells to contribute to leaky gut [51]. In order to better study the regulation of PHP or other prebiotics on gut microbiota, metagenomic sequencing, short-chain fatty acid, and the determination of other metabolites need to be introduced in further study. These abovementioned results proposed that PHP might relieve gut inflammation by adjusting several key microorganisms to augment the content of short-chain fatty acids and to improve intestinal barrier damage.

## 4. Materials and Methods

### 4.1. Materials and Reagents

DSS, molecular weight (MW) of 36–50 kDa, was obtained from MP Biochemicals (Santa Ana, CA, USA). Paraformaldehyde Fix Solution (4%), antibodies against zonula occludens-1 (ZO-1, GB111402) and Claudin-1 (GB112523) were purchased from Sevicebio Technology Co., Ltd. (Wuhan, China). Antibodies against Occludin (A12621) and β-actin (AC026) were purchased from ABclonal Technology Co., Ltd. (Wuhan, China). CD3 (100203), CD4 (100407), CD25 (102010), IL-17A (506915), Foxp3 (126407), Cell Activation Cocktail (with Brefeldin A, phorbol 12-myristate 13-acetate (PMA), ionomycin, etc.) (423303) and True-Nuclear^TM^ 4X Fix Concentrate (B337252) were purchased from BioLegend (San Diego, CA, USA).

### 4.2. Preparation and Identification of Polysaccharide

Crude PHP (S27812) was extracted from the fermentation broth of *Paecilomyces hepiali* that purchased from Shanghai Yuanye Bio-Technology Co., Ltd. (Shanghai, China). The crude PHP solution was repeatedly deproteinated by the sevage method [18] and purified by ethanol precipitation [52]. The molecular size distribution of PHP was analyzed by Agilent 1260 LC instrument (Agilent Technologies, Santa Clara, CA, USA) with a TSKgel GMPW_XL_ analysis column (7.8 mm × 300 mm, Tosoh, Tokyo, Japan). The MW of the PHP was calculated according to the calibration curve established by the dextran standards (T-10, T-40, T-70, T-500, and T-2000). The monosaccharide composition of PHP was analyzed by high-performance liquid chromatography (HPLC) via Agilent 1260 LC instrument (Agilent Technologies Inc, Santa Clara, CA, USA) with a ZORBAX Eclipse XDB-C18 column (id 5 µm, 4.6 × 250 mm, Agilent Technologies Inc., Santa Clara, CA, USA) according to the monosaccharide standard, including rhamnose, arabinose and galactose, etc. The Fourier transforms infrared spectroscopy (FT-IR) spectrum of PHP was detected with a FT-IR spectrometer (Alpha type, Bruker, Billerica, MA, USA). PHP was processed by KBr pressure disk technology and measured within the frequency range of 4000~400 cm^−1^.

### 4.3. Animals and Induction of Colitis

C57BL/6J male mice (16–18 g) were purchased from Jinan Pengyue Experimental Animal Breeding Co., Ltd. (Jinan, China) and housed at SPF conditions. After 2 weeks of adaptation, all mice were randomly divided into the three experimental groups, and received the different treatments as in Figure 2A: (1) NC group: no treatment for all 3 weeks (n = 6); (2) DSS group (n = 7): 2.5% DSS for the last week; (3) PHP group (n = 6): PHP (400 mg/kg/day) for all 3 weeks, and 2.5% DSS for the last week. Colitis was induced in mice with free drinking water containing 2.5% DSS.

All the mice were weighed daily. Fresh feces were collected and kept at −80 °C for gut microbiota analysis. All the mice were anesthetized before serum samples were collected. After euthanasia, the colon samples were obtained, and their lengths were identified. The colon samples were stored at −80 °C for RNA/protein extraction and histological analyses. Simultaneously, the spleens of mice were gathered for flow cytometry analysis. All animal procedures were approved by the Ethics Committee of the Medical College of Qingdao University (QDU-AEC-2022359). All animal experiments should comply with the ARRIVE guidelines [53].

### 4.4. Histological Evaluation

The colon samples were fixed and embedded with paraffin. The above colon tissue sections with a thickness of 4 μm were subjected to alcian blue and hematoxylin and eosin (H&E) staining as previously depicted [54]. Additionally, the histological scoring method was determined as previously described [55].

### 4.5. Western Blot (WB) Analysis

The cold RIPA lysis buffer (Solarbio, Beijing, China) was applied to extract the total protein from colon tissue samples. Protein concentration was determined by using the BCA assay kit (Epizyme, Shanghai, China). Sodium dodecyl sulfate polyacrylamide gel electrophoresis (SDS-PAGE) was used to separate an equal number of denatured proteins. Then, the proteins were transferred to PVDF membrane (Millipore, Billerica, MA, USA), and the membrane was blocked with 1X Protein Free Rapid Blocking Buffer (Epizyme, Shanghai, China). After incubation with primary antibodies and secondary antibodies, the protein signals were detected with Ultra High Sensitivity ECL Kit (GlpBio, Montclair, CA, USA).

### 4.6. RNA Extraction and RT-qPCR

Total RNA was extracted from colon tissues by RNA Extraction Kit (SparkJade, Jinan, China). Then, cDNAs were produced by SPARK script II RT Plus Kit (SparkJade, Jinan, China). Data obtained through RT-qPCR using kit from SparkJade (Jinan, China) were analyzed by 2^−ΔΔCt^ method. *GAPDH* gene expression was used to standardize gene expression levels. The primer sequences were listed in Table S1.

### 4.7. Cytokine Measurements

Whole blood samples were gathered and centrifuged at 3000 rpm for 15 min to obtain serum. All the supernatants were collected and stored at −80 °C for enzyme-linked immunosorbent assay (ELISA) detection. The concentration of proinflammatory cytokines of serum was determined via the ELISA kit (Abclonal, Wuhan, China).

### 4.8. Flow Cytometry

Primary spleen cells of mice were isolated as described in the previous study [56]. In short, the spleens of mice were obtained under aseptic conditions. After grinding, the red blood cells were lysed and obtained single cell suspension. The specific method for measuring the number of T helper type 17 (Th17) cells and regulatory T (Treg) cells via flow cytometry referred to our previous study [57].

### 4.9. Gut Microbiota Analysis

The DNA of gathered fecal samples were abstracted for PCR amplification and 16S RNA sequencing based on a previous description [58]. Subsequently, data analysis method of gut microbiota distribution and structure were from our previous study [58].

### 4.10. Database Mining

Transcriptome data (GSE87466) of normal colon tissues and colitis tissues were downloaded from the Gene Expression Omnibus (GEO) database [59] (https://www.ncbi.nlm.nih.gov/geo/, accessed on 1 December 2018). The list of genes related to Treg and Th17 signaling pathways were summarized from the Kyoto Encyclopedia of Genes and Genomes (KEGG) database (https://www.genome.jp/kegg/, accessed on 1 December 2018). The expression quantity was transformed into log_2_ and then the R software was used to construct the heat map.

### 4.11. Statistical Analysis

Statistical analysis was completed with GraphPad Prism 9.3 (La Jolla, CA, USA). Significant differences were evaluated by one-way the analysis of variance (ANOVA) for multiple groups and Students’ *t*-test for two groups, and * *p* < 0.05, ** *p* < 0.01, *** *p* < 0.001 were regarded as statistically significant.

## 5. Conclusions

Taken together, our findings demonstrate PHP has the ability to ameliorate DSS-induced colitis symptoms in mice. The beneficial effect of PHP was mainly mediated via ameliorating microbiota dysbiosis, promoting intestinal barrier functions, positively regulating inflammatory factors and Treg/Th17 cell balance. These findings provide a potential insight into prevention and treatment of UC.

## Data Availability

The original contributions presented in the study are publicly available. This data can be found here: (https://www.ncbi.nlm.nih.gov/) SUB12938033, accessed on 1 May 2023.

## Supplementary Materials

The following supporting information can be downloaded at: https://www.mdpi.com/article/10.3390/molecules28134984/s1, Table S1: Primers used for RT-qPCR.

## Sample Availability

Not available.

## References

[1] Lasa J.S., Olivera P.A., Danese S., Peyrin-Biroulet L. (2022). Efficacy and safety of biologics and small molecule drugs for patients with moderate-to-severe ulcerative colitis: A systematic review and network meta-analysis. Lancet Gastroenterol. Hepatol..

[2] Kaplan G.G. (2015). The global burden of IBD: From 2015 to 2025. Nat. Rev. Gastroenterol. Hepatol..

[3] Monstad I.L., Solberg I.C., Cvancarova M., Hovde O., Henriksen M., Huppertz-Hauss G., Gunther E., Moum B.A., Stray N., Vatn M. (2021). Outcome of Ulcerative Colitis 20 Years after Diagnosis in a Prospective Population-based Inception Cohort from South-Eastern Norway, the IBSEN Study. J. Crohn’s Colitis.

[4] Grossberg L.B., Papamichael K., Cheifetz A.S. (2022). Review article: Emerging drug therapies in inflammatory bowel disease. Aliment. Pharmacol. Ther..

[5] Caio G., Lungaro L., Chiarioni G., Giancola F., Caputo F., Guarino M., Volta U., Testino G., Pellicano R., Zoli G. (2022). Beyond biologics: Advanced therapies in inflammatory bowel diseases. Minerva Gastroenterol..

[6] Ahlawat S., Kumar P., Mohan H., Goyal S., Sharma K.K. (2021). Inflammatory bowel disease: Tri-directional relationship between microbiota, immune system and intestinal epithelium. Crit. Rev. Microbiol..

[7] de Vos W.M., Tilg H., Van Hul M., Cani P.D. (2022). Gut microbiome and health: Mechanistic insights. Gut.

[8] Leonardi I., Gao I.H., Lin W.Y., Allen M., Li X.V., Fiers W.D., De Celie M.B., Putzel G.G., Yantiss R.K., Johncilla M. (2022). Mucosal fungi promote gut barrier function and social behavior via Type 17 immunity. Cell.

[9] Chang J.T. (2020). Pathophysiology of Inflammatory Bowel Diseases. N. Engl. J. Med..

[10] Radziszewska M., Smarkusz-Zarzecka J., Ostrowska L., Pogodzinski D. (2022). Nutrition and Supplementation in Ulcerative Colitis. Nutrients.

[11] Keshteli A.H., Madsen K.L., Dieleman L.A. (2019). Diet in the Pathogenesis and Management of Ulcerative Colitis; A Review of Randomized Controlled Dietary Interventions. Nutrients.

[12] Niu W., Chen X., Xu R., Dong H., Yang F., Wang Y., Zhang Z., Ju J. (2021). Polysaccharides from natural resources exhibit great potential in the treatment of ulcerative colitis: A review. Carbohydr. Polym..

[13] Yu B., Wang M., Teng B., Veeraperumal S., Cheung P.C., Zhong S., Cheong K.L. (2023). Partially Acid-Hydrolyzed Porphyran Improved Dextran Sulfate Sodium-Induced Acute Colitis by Modulation of Gut Microbiota and Enhancing the Mucosal Barrier. J. Agric. Food Chem..

[14] Dong C., Guo S., Wang W., Liu X. (2015). Cordyceps industry in China. Mycology.

[15] Yang S., Yang X., Zhang H. (2020). Extracellular polysaccharide biosynthesis in Cordyceps. Crit. Rev. Microbiol..

[16] Li X., Liu Q., Li W., Li Q., Qian Z., Liu X., Dong C. (2019). A breakthrough in the artificial cultivation of Chinese cordyceps on a large-scale and its impact on science, the economy, and industry. Crit. Rev. Biotechnol..

[17] Wang J., Li L.Z., Liu Y.G., Teng L.R., Lu J.H., Xie J., Hu W.J., Liu Y., Liu Y., Wang D. (2016). Investigations on the antifatigue and antihypoxic effects of Paecilomyces hepiali extract. Mol. Med. Rep..

[18] Hu W., Wang J., Guo W., Liu Y., Guo Z., Miao Y., Wang D. (2020). Studies on characteristics and anti-diabetic and -nephritic effects of polysaccharides isolated from Paecilomyces hepiali fermentation mycelium in db/db mice. Carbohydr. Polym..

[19] Wu Z., Lu J., Wang X., Hu B., Ye H., Fan J., Abid M., Zeng X. (2014). Optimization for production of exopolysaccharides with antitumor activity in vitro from Paecilomyces hepiali. Carbohydr. Polym..

[20] Wu Z., Zhang M., Xie M., Dai Z., Wang X., Hu B., Ye H., Zeng X. (2016). Extraction, characterization and antioxidant activity of mycelial polysaccharides from Paecilomyces hepiali HN1. Carbohydr. Polym..

[21] Ma L., Chen H., Zhang Y., Zhang N., Fu L. (2012). Chemical modification and antioxidant activities of polysaccharide from mushroom Inonotus obliquus. Carbohydr. Polym..

[22] Yan L., Xiong C., Xu P., Zhu J., Yang Z., Ren H., Luo Q. (2019). Structural characterization and in vitro antitumor activity of A polysaccharide from *Artemisia annua* L. (Huang Huahao). Carbohydr. Polym..

[23] Huang Y., Gao Y., Pi X., Zhao S., Liu W. (2022). In Vitro Hepatoprotective and Human Gut Microbiota Modulation of Polysaccharide-Peptides in Pleurotus citrinopileatus. Front. Cell. Infect. Microbiol..

[24] Copikova J., Synytsya A., Novethna M. (2001). Application of FT-IR Spectroscopy in Detection of Food Hydrocolloids in Confectionery Jellies and Food Supplements. Czech J. Food Sci..

[25] Neurath M.F. (2014). Cytokines in inflammatory bowel disease. Nat. Rev. Immunol..

[26] Paone P., Cani P.D. (2020). Mucus barrier, mucins and gut microbiota: The expected slimy partners?. Gut.

[27] Lai H., Yang Z., Lou Z., Li F., Xie F., Pan W., Xu C., Zhang L., Zhang S., Zhang L. (2021). Root Extract of Lindera aggregata (Sims) Kosterm. Modulates the Th17/Treg Balance to Attenuate DSS-Induced Colitis in Mice by IL-6/STAT3 Signaling Pathway. Front. Pharmacol..

[28] Zhang W., Cheng C., Han Q., Chen Y., Guo J., Wu Q., Zhu B., Shan J., Shi L. (2019). Flos Abelmoschus manihot extract attenuates DSS-induced colitis by regulating gut microbiota and Th17/Treg balance. Biomed. Pharmacother..

[29] Xiao J.H., Qi Y., Xiong Q. (2013). Nucleosides, a valuable chemical marker for quality control in traditional Chinese medicine Cordyceps. Recent Pat. Biotechnol..

[30] Li Y.Y., Wang X.J., Su Y.L., Wang Q., Huang S.W., Pan Z.F., Chen Y.P., Liang J.J., Zhang M.L., Xie X.Q. (2022). Baicalein ameliorates ulcerative colitis by improving intestinal epithelial barrier via AhR/IL-22 pathway in ILC3s. Acta Pharmacol. Sin..

[31] Fang J., Wang H., Zhou Y., Zhang H., Zhou H., Zhang X. (2021). Slimy partners: The mucus barrier and gut microbiome in ulcerative colitis. Exp. Mol. Med..

[32] Turner J.R. (2009). Intestinal mucosal barrier function in health and disease. Nat. Rev. Immunol..

[33] Kaser A., Zeissig S., Blumberg R.S. (2010). Inflammatory bowel disease. Annu. Rev. Immunol..

[34] Kaminsky L.W., Al-Sadi R., Ma T.Y. (2021). IL-1β and the Intestinal Epithelial Tight Junction Barrier. Front. Immunol..

[35] Fan L., Qi Y., Qu S., Chen X., Li A., Hendi M., Xu C., Wang L., Hou T., Si J. (2021). *B. adolescentis* ameliorates chronic colitis by regulating Treg/Th2 response and gut microbiota remodeling. Gut Microbes.

[36] Britton G.J., Contijoch E.J., Mogno I., Vennaro O.H., Llewellyn S.R., Ng R., Li Z., Mortha A., Merad M., Das A. (2019). Microbiotas from Humans with Inflammatory Bowel Disease Alter the Balance of Gut Th17 and RORγt(+) Regulatory T Cells and Exacerbate Colitis in Mice. Immunity.

[37] Liu Y.J., Tang B., Wang F.C., Tang L., Lei Y.Y., Luo Y., Huang S.J., Yang M., Wu L.Y., Wang W. (2020). Parthenolide ameliorates colon inflammation through regulating Treg/Th17 balance in a gut microbiota-dependent manner. Theranostics.

[38] Jonuleit H., Schmitt E. (2003). The regulatory T cell family: Distinct subsets and their interrelations. J. Immunol..

[39] Zeng H., Chi H. (2015). Metabolic control of regulatory T cell development and function. Trends Immunol..

[40] Lee S.Y., Lee S.H., Yang E.J., Kim E.K., Kim J.K., Shin D.Y., Cho M.L. (2015). Metformin Ameliorates Inflammatory Bowel Disease by Suppression of the STAT3 Signaling Pathway and Regulation of the between Th17/Treg Balance. PLoS ONE.

[41] Wang Y., Zhang J., Xu L., Ma J., Lu M., Ma J., Liu Z., Wang F., Tang X. (2021). Modified Gegen Qinlian Decoction Regulates Treg/Th17 Balance to Ameliorate DSS-Induced Acute Experimental Colitis in Mice by Altering the Gut Microbiota. Front. Pharmacol..

[42] Cui H., Cai Y., Wang L., Jia B., Li J., Zhao S., Chu X., Lin J., Zhang X., Bian Y. (2018). Berberine Regulates Treg/Th17 Balance to Treat Ulcerative Colitis through Modulating the Gut Microbiota in the Colon. Front. Pharmacol..

[43] Ni J., Wu G.D., Albenberg L., Tomov V.T. (2017). Gut microbiota and IBD: Causation or correlation?. Nat. Rev. Gastroenterol. Hepatol..

[44] Wallace B.D., Wang H., Lane K.T., Scott J.E., Orans J., Koo J.S., Venkatesh M., Jobin C., Yeh L.A., Mani S. (2010). Alleviating cancer drug toxicity by inhibiting a bacterial enzyme. Science.

[45] Kalyuzhnaya M.G., Lapidus A., Ivanova N., Copeland A.C., McHardy A.C., Szeto E., Salamov A., Grigoriev I.V., Suciu D., Levine S.R. (2008). High-resolution metagenomics targets specific functional types in complex microbial communities. Nat. Biotechnol..

[46] Derrien M., Belzer C., de Vos W.M. (2017). Akkermansia muciniphila and its role in regulating host functions. Microb. Pathog..

[47] Flint H.J., Bayer E.A., Rincon M.T., Lamed R., White B.A. (2008). Polysaccharide utilization by gut bacteria: Potential for new insights from genomic analysis. Nat. Rev. Microbiol..

[48] Machiels K., Joossens M., Sabino J., De Preter V., Arijs I., Eeckhaut V., Ballet V., Claes K., Van Immerseel F., Verbeke K. (2014). A decrease of the butyrate-producing species *Roseburia hominis* and *Faecalibacterium prausnitzii* defines dysbiosis in patients with ulcerative colitis. Gut.

[49] Chen Y., Liu Y., Wang Y., Chen X., Wang C., Chen X., Yuan X., Liu L., Yang J., Zhou X. (2022). Prevotellaceae produces butyrate to alleviate PD-1/PD-L1 inhibitor-related cardiotoxicity via PPARα-CYP4X1 axis in colonic macrophages. J. Exp. Clin. Cancer Res. CR.

[50] Lu Y., Yuan X., Wang M., He Z., Li H., Wang J., Li Q. (2022). Gut microbiota influence immunotherapy responses: Mechanisms and therapeutic strategies. J. Hematol. Oncol..

[51] Singh S.B., Coffman C.N., Varga M.G., Carroll-Portillo A., Braun C.A., Lin H.C. (2022). Intestinal Alkaline Phosphatase Prevents Sulfate Reducing Bacteria-Induced Increased Tight Junction Permeability by Inhibiting Snail Pathway. Front. Cell. Infect. Microbiol..

[52] Hao W., Chen Z., Yuan Q., Ma M., Gao C., Zhou Y., Zhou H., Wu X., Wu D., Farag M.A. (2022). Ginger polysaccharides relieve ulcerative colitis via maintaining intestinal barrier integrity and gut microbiota modulation. Int. J. Biol. Macromol..

[53] McGrath J.C., Lilley E. (2015). Implementing guidelines on reporting research using animals (ARRIVE etc.): New requirements for publication in BJP. Br. J. Pharmacol..

[54] He N., Wang Y., Zhou Z., Liu N., Jung S., Lee M.S., Li S. (2021). Preventive and Prebiotic Effect of α-Galacto-Oligosaccharide against Dextran Sodium Sulfate-Induced Colitis and Gut Microbiota Dysbiosis in Mice. J. Agric. Food Chem..

[55] Peng Y., Yan Y., Wan P., Chen D., Ding Y., Ran L., Mi J., Lu L., Zhang Z., Li X. (2019). Gut microbiota modulation and anti-inflammatory properties of anthocyanins from the fruits of Lycium ruthenicum Murray in dextran sodium sulfate-induced colitis in mice. Free Radic. Biol. Med..

[56] Sendler M., van den Brandt C., Glaubitz J., Wilden A., Golchert J., Weiss F.U., Homuth G., De Freitas Chama L.L., Mishra N., Mahajan U.M. (2020). NLRP3 Inflammasome Regulates Development of Systemic Inflammatory Response and Compensatory Anti-Inflammatory Response Syndromes in Mice With Acute Pancreatitis. Gastroenterology.

[57] Zhou Z., Yu S., Cui L., Shao K., Pang H., Wang Z., He N., Li S. (2022). Isomaltulose alleviates acute colitis via modulating gut microbiota and the Treg/Th17 balance in mice. Food Funct..

[58] Wang H., Liu N., Yang Z., Zhao K., Pang H., Shao K., Zhou Z., Li S., He N. (2022). Preventive effect of pectic oligosaccharides on acute colitis model mice: Modulating epithelial barrier, gut microbiota and Treg/Th17 balance. Food Funct..

[59] Li K., Strauss R., Ouahed J., Chan D., Telesco S.E., Shouval D.S., Canavan J.B., Brodmerkel C., Snapper S.B., Friedman J.R. (2018). Molecular Comparison of Adult and Pediatric Ulcerative Colitis Indicates Broad Similarity of Molecular Pathways in Disease Tissue. J. Pediatr. Gastroenterol. Nutr..

