# Supplementary material for: Polysaccharides from Paecilomyces hepiali Prevent Acute Colitis in Association with Modulating Gut Microbiota and Treg/Th17 Immune Balance in Mice
Source: Molecules. 2023 Jun 25;28(13):4984. doi: 10.3390/molecules28134984 (PMC10343787; doi:10.3390/molecules28134984)
Supplement: Supplementary file 1 [file molecules-28-04984-s001.zip › molecules-2466546-supplementary.pdf]

Supplementary Table S1. Primers used for RT-qPCR

Gene name	Primer's sequence
GAPDH	F:GGTTGTCTCCTGCGACTTCA<br>R:TGGTCCAGGGTTTCTTACTCC
IL-10	F:GGTTGTCGTCTCATTCTGAAAGA<br>R:GGTAGAGGACCCAAGTTCGTTAAGA
IL-6	F:CTTCTTGGGACTGATGCTGGTGAC<br>R:AGGTCTGTTGGGAGTGGTATCCTC
TNFα	F:ATGTCTCAGCCTCTTCTCATTC<br>R:GCTTGTCACTCGAATTTTGAGA
IL-1β	F:TCGCAGCAGCACATCAACAAGAG<br>R:AGGTCCACGGGAAAGACACAGG
ZO-1	F:CTGGTGAAGTCTCGGAAAAATG<br>R:CATCTCTTGCTGCCAAACTATC
Claudin-1	F:AGATACAGTGCAAAGTCTTCGA<br>R:CAGGATGCCAATTACCATCAAG
Occludin	F:TGCTTCATCGCTTCCTTAGTAA<br>R:GGGTTCACTCCCATTATGTACA

F: Forword; R: Reverse
